# New York Letter

**Published:** 1893-06

**Authors:** Wm. L. Russell

**Affiliations:** 151 East 50th St.; New York


### *CORRESPONDENCE.*

### NEW YORK LETTER.

New York, May 15, 1893.

PILOCARPINE.

A valuable contribution to the knowledge of the physiological action of this remedy was made in a paper read at the last meeting of the Academy by Dr. W. Prentiss, of Washington, D. C. A remarkable action of the drug in changing the color of the hair had been observed by him in a case in which it had been administered as a sudorific.

The case was that of a female, aged twenty-five, a blonde, who was affected with pyelonephritis of twenty-one days standing, during which there had been almost complete anuria. The effect of the remedy was an immediate reddening of the face and neck, accompanied by palpitation and nausea. This was followed in a few minutes by salivation, and soon afterwards by profuse sweating, so that drops of water ran from the body, and the hair of the patient and the bedding were saturated. A urinous odor was noticeable.

Before salivation began water flowed freely from the eyes and nose. This discharge ceased, however, when salivation was established. Vomiting was severe and exhausting, continuing throughout the period of the action of the remedy. The odor of the vomited matter was offensive, being similar to that of decaying vegetable matter. The bowels moved two or three times, peristalsis being excited, and an increased quantity of liquid being discharged. He estimated that about fourteen pounds of fluid was lost by the patient in six hours.

The pulse during this period became rapid, weak and compressible, its rate varying from 120 to 136. There was thumping palpitation of the heart which was distressing to the patient, and could be heard at some distance from her.

The sight was dim, and the pupils contracted to a point. The patient showed a disposition to sleep, and experienced a sense of relief when diaphoresis was established. A good recovery followed under this treatment, continued for ten days.

At the end of this period, it was found that the hair, which at the beginning of the treatment was fine and of a pale yellow color, had become quite coarse and dark brown in color. Specimens of the hair taken during the process of change were shown by Dr. Prentiss, and presented a striking appearance.

In addition to the actions of the pilocarpine noted in the case described, Dr. Prentiss stated that it caused an increase in the mucous secretion of the bronchi and of the intestines. It also increased the urinary secretion, but not that of bile. The amount of urea eliminated by the skin was increased very largely. Its action in the bronchial mucous membrane might be utilized in the treatment of diphtheria were it not for the depressing effect on the heart. When bronchitis and œdema of the lungs existed, the profuse secretion caused might result in suffocation of the patient. If the dropsy were due to cardiac disease, it would be necessary to administer stimulants, simultaneously, with the remedy.

Atropine antagonizes the action of pilocarpine in some particulars. It counteracted its effect on the pupil and stopped the sweating. In some other respects, however, the actions of these remedies were similar. The same frontal headache and pain in the bladder occurred by the use of each agent, and they also agreed in affecting children proportionately less than adults.

In a case of diphtheria in which the remedy was used, the hair was darkened, but the color faded a few days afterwards. In a man, aged seventy-two, who had contracted kidneys, the eyebrows and hair grew considerably darker. These changes in the color of the hair were difficult to explain, but were probably akin to that caused by certain articles of food in the lower animals. It was well-known that environment had an influence in the coloration of birds and animals, and the effect of diet was seen in the dark yellow color produced in canaries by feeding them with cayenne pepper, and the coloring of the bones and of the plumage of birds by madder, and perhaps by certain shell-fish.

CONVULSIONS IN CHILDREN.

This subject was discussed at the last meeting of the Section on Pædiatrics. Dr. Landon Carter Gray spoke about the differential diagnosis of the various forms of convulsions. He stated that convulsions due to organic lesions, such as meningitis, encephalitis, and hæmorrhage, were best diagnosed by the mental defects which would be noticed to follow them. Convulsions due to peripheral irritation most frequently had their origin in putrefaction of food in the alimentary canal. They were seldom or never caused by genital irritation.

Dr. H. W. Berg said in regard to the pathology and treatment of simple eclampsia in children, that the cases could be divided into three classes: those in which the convulsions were due to high fever, those in which they were of reflex origin, and those in which they were toxæmic. The primary irritation producing a convulsion might be in any portion of the nervous system, but the real starting point was the medulla. The mechanism regulating the heat-loss and heat-distribution of the body was in the medulla also, and was in close contact with the motor fibres passing through it. Disturbance of this center might thus act as a factor in causing a convulsion. The rise of temperature in status epilepticus might perhaps be explained by this anatomical fact.

The first step in treatment consisted in making a differential diagnosis. His custom was to first take the temperature. If this were found above 102°, he used antipyretic measures and was often enabled in this way to dispense with intispasmodics. Sponging with alcohol one part and water three parts was employed, and antipyrine in three to six grain doses by mouth or rectum. An enema was given, and an ice-pack applied to the head. In reflex cases the stomach and rectum should be emptied. In toxic cases a hot-pack, a mustard bath and diuretics should be employed.

Dr. G. M. Hammond discussed the effect of early diagnosis and treatment in preventing after-effects. He said that the importance of this matter became apparent in the statement of Gowers, that one-eighth of all the cases of epilepsy began during the first three years of life. *Petit mal* was frequently overlooked in young children, and it would be well for the physician to consider the possibility of its existence in cases giving a history of frequent falls without apparent cause. Parents were usually entirely ignorant of the possibility of such a condition, and paid little attention to these mild attacks until signs of stupidity or uncleanly habits drew their attention to the mental defects in the child. Epilepsy had a tendency to become organic in the delicate growing brain of a young child.

Convulsive attacks were never trivial, and when a child had already had one, preventive measures should be adopted. A reflex irritation in a nervous system defective by heredity was the usual cause. It was therefore important that the physical development of such a child should be carefully attended to. It was foolish to push the intellectual development of these children at the expense of physical robustness. If epilepsy was already present, the diet became an important feature in treatment. The digestive organs were always sluggish in epilepsy, and careful management of the food was necessary. The quantity of food should be regulated. Milk should enter largely into the dietary; it might be peptonized. Digested proteids were also valuable. Medicinally, no remedy had yet been discovered that was so universally useful as the bromides. They should be given in doses just large enough to prevent the occurrence of the paroxysms, no larger, and should be continued for one and a half or two years after the paroxysms had ceased. The majority of cases could be cured, if taken early. Dr. Walter L. Carr believed that rachitis was a frequent cause, especially because of the catarrhs of the digestive tract so prevalent in cases of that disease. The so-called febrile cases he thought were of toxic origin.

Dr. Downing was of the opinion that teething seldom operated as a cause of convulsions, nor did he think that fever was important unless it reached 104°. His treatment consisted in the use of chloroform, if the case was urgent, and of chloral.

Dr. Chapin said that he had noticed that in cases of simple eclampsia there was usually some rise of temperature while in epilepsy there was none.

Prof. J. Lewis Smith said that his long experience at Bellevue had shown him that most cases of epilepsy could be traced back to eclampsia in childhood. He believed then that early treatment might prove to be preventive. He had treated many cases of convulsions before bromide and chloral were added to our therapeutic resources, and had often remained with a case as long as twelve hours. Since the introduction of these remedies the duration of these cases was much shorter. Sixty grains of bromide were given in one dose to a child a year and a half old, by mistake, with the effect of quieting the convulsion, and without injury to the child. He also used chloral, in doses of three to five grains by rectum, in a child of a year old.

### EMPYEMA.

At the same meeting, in discussion drawn forth by the presentation of acase of recovered empyema, the consensus of opinion seemed to be opposed to the employment of irrigation after operation. Dr. Koplik advocated the resection of a portion of the rib, better drainage being secured, and rapid recovery being promoted.

151 *East 50th St.* WM. L. RUSSELL.

---

### HEART FAILURE.

Prof. Alfred L. Loomis, of New York, discusses this question in a paper read before the American Climatological Association and printed in a recent number of the *International Medical Magazine*. Dr. Loomis includes all heart failures in three classes: (1) Those in which the heart has for a long time been called upon to perform an abnormal amount of work, as in valvular or arterial disease. (2) Those in which obstructive changes in the coronary vessels markedly diminish the nutritive supply of the cardiac muscle. (3) Those in which toxic influences act directly upon the nutrition of the cardiac muscle, or so interfere with the cardiac nerve-supply as to lessen cardiac resistance. The paper concludes as follows: "A review of the cases which I have presented makes it evident that the term heart failure is misleading and should be abandoned, for in most instances it does not express the pathological state. It is equally evident that the term 'death by heart failure' is often used to cover the ignorance of medical attendants."—*Nat. Pop. Review.*

